# Early Assessment of Neurodevelopmental Scales Correlates With Positive M-CHAT Screening in 24-Month-Old Infants

**DOI:** 10.31083/AP42936

**Published:** 2026-03-24

**Authors:** Haixia Chang, Yali Liu, Jinfang Lv, Qin Li, Junqiang Xue, Xia Wang

**Affiliations:** ^1^Pediatric Health Care Department & Psychological Behavior Department, Hebei Children's Hospital, 050031 Shijiazhuang, Hebei, China; ^2^Hebei Provincial Clinical Research Center for Child Health and Disease, 050031 Shijiazhuang, Hebei, China; ^3^Department of Respiratory Medicine, Hebei Children's Hospital, 050031 Shijiazhuang, Hebei, China; ^4^Department of Vascular Surgery, Shijiazhuang People's Hospital, 050000 Shijiazhuang, Hebei, China

**Keywords:** autism spectrum disorder, child development, mass screening, motor skills, neurodevelopmental disorders, neuropsychological tests

## Abstract

**Background:**

Autism spectrum disorder (ASD) is a neurodevelopmental condition characterized by deficits in social communication and repetitive behaviors. Early identification of high-risk infants is crucial for intervention and long-term outcomes. This study explored the association between early neurological development from 12 to 15 months and the related risk factors for ASD, with a primary focus on M-CHAT status at 24 months as the screening outcome, and the impact of family history, prematurity, and sleep disorders.

**Methods:**

We conducted a retrospective analysis of 50 children aged 24 months who tested positive on the Modified Checklist for Autism in Toddlers (M-CHAT) and 50 matched controls with negative results. Neurodevelopmental status was assessed using the Gesell Developmental Diagnosis Scale (GDDS), the Peabody Developmental Motor Scales, Second Edition (PDMS-2), and the Sensory Integration Schedule (SIS). Statistical analyses, including logistic regression, were used to evaluate associations among baseline characteristics, neurodevelopmental scores, and ASD risk factors.

**Results:**

A total of 100 infants were included, with 50 classified as high risk and 50 as low risk. High-risk infants had significantly higher rates of family history of ASD, prematurity, and sleep disorders. They showed impairments across several PDMS-2 subscales, including stationary, locomotion, and grasping, as well as GDDS subscales such as adaptability and language. Logistic regression analysis revealed that neurodevelopmental impairments were significantly associated with M-CHAT positivity at 24 months.

**Conclusions:**

Early neurodevelopmental impairments in infants aged 12 to 15 months may serve as important indicators of ASD risk. Identifying these factors may facilitate timely intervention and improve outcomes for high-risk children.

## Main Points

1. Infants who screen positive for autism risk at 24 months already demonstrate significant neurodevelopmental impairments in motor, language, and social domains as early as 12 to 15 months of age.

2. Specific motor skills, particularly grasping and stationary stability, along with language development and adaptability, serve as key early indicators of later positive Modified Checklist for Autism in Toddlers (M-CHAT) screening results.

3. Factors such as a family history of autism, premature birth, and early childhood sleep disorders are strongly associated with a higher risk of neurodevelopmental delays.

4. Early objective assessment using standardized scales like Peabody Developmental Motor Scales-2 (PDMS-2) and Gesell Developmental Diagnosis Scale (GDDS) can help identify high-risk infants before traditional screening ages, facilitating timely and targeted interventions.

## 1. Introduction

Autism spectrum disorder (ASD) is a broad neurodevelopmental condition characterized primarily by deficits in social communication, limited interests, and repetitive or stereotyped behaviors [1, 2]. Children diagnosed with ASD often have higher instances of co-occurring medical issues, including intellectual disability and attention deficit hyperactivity disorder [3]. In recent years, there has been a significant rise in the global prevalence of ASD. An epidemiological study conducted in the United States in 2021 indicated that the rate of ASD was 23 cases per 1000 among children who are 8 years old [2]. This surge in ASD prevalence has attracted considerable attention from healthcare and educational professionals, as well as parents and the wider public.

Early identification and prediction of ASD development is vital for improving the prognosis of these high-risk infants [1]. Several reports have developed models to predict the occurrence of ASD, with a focus on using the Modified Checklist for Autism in Toddlers (M-CHAT) as a screening tool for 24-month-old children. However, these techniques require comprehensive laboratory examinations or electroencephalography [4, 5]. Widespread screening of infants is difficult. Screening of ASD subjects is complicated due to their impaired areas of social and cognitive capabilities and levels of functioning. Notably, recent developments have suggested that a significant number of children with ASD display neurodevelopmental setbacks during their first or second years, including delays in language and social skills, along with motor development issues [6]. Most children with ASD who are under 5 years of age experience a combined global developmental delay, which may be evidenced by delays across two or more areas of development. What is more important is that for infants as young as 12–15 months, neurocognitive markers help predict the occurrence of ASD [7]. Therefore, for children with high-risk of ASD, early detection of these neurodevelopmental delays might help identify and predict the occurrence, and lead to early intervention. In addition, a number of other assessment tools were created to comprehensively represent the neurodevelopmental condition, including the Gesell Developmental Diagnosis Scale (GDDS), the Sensory Integration Schedule (SIS), and the Peabody Developmental Motor Scales (PDMS).

ASD is thought to arise from a mix of genetic and environmental influences. We selected family history (genetic), prematurity, and sleep disorders (environmental) based on: (a) strong epidemiological association (family history OR = 7.4 [8]; prematurity OR = 2.5 [9]; or sleep-disorder prevalence 50–80% in ASD [10]); (b) biological plausibility in neurodevelopment; (c) clinical feasibility for early screening. These factors represent core etiological pathways: genetic vulnerability (family history), perinatal compromise (prematurity), and postnatal neural dysfunction (sleep disorders). Consistent with this framework, current evidence has confirmed that various risk factors are strongly associated with heightened ASD risk. For example, an increasing body of evidence has suggested that prenatal experiences and the health of the mother during pregnancy can have enduring impacts on the neurodevelopment of children [11]. Parental rearing pattern [12] and nutritional status [13] have also been reported to be potential risk factors. However, the relationship between these risk factors and the neurodevelopmental delays is not quite clear, especially in children at high risk for ASD. Therefore, we hypothesized that the early neurological developmental patterns at 12-months to 15-months of age, in combination with key genetic or environmental factors, were significantly correlated with the onset risk of ASD. We retrospectively analyzed the key early neurodevelopmental patterns of infants (between 12-month- and 15-month-old) with high-risk for ASD at 24 months, matched with low-risk ASD infants born in the same month. The possible risk factors were also analyzed to reveal the possible contribution to these neurodevelopmental disorders and ASD progression. Our study may provide early clues of neurodevelopmental disorders for these children.

## 2. Materials and Methods

### 2.1 Participants

First, the sample size was estimated using the MedSci Sample Size Tools (MSST, MedSci, Shanghai, China) according to “Sample Size Tables for Clinical Studies, 2nd Ed.”. The calculation assumed a large effect size (Cohen’s d = 0.8) based on neurodevelopmental score differences reported in previous ASD studies [14, 15, 16], with α at *p* ≤ 0.05 (two-tailed) and 80% power, and an allocation ratio of 1:1. This yielded a required sample of 40/gp. To account for potential attrition and increase robustness for subgroup analyses, we expanded to 49/gp (total *n* = 98). Approval for this study was obtained from the Ethics Committee at Hebei Children’s Hospital affiliated to Hebei Medical University (No. 202136). During the study period (July 2022 to December 2023), 520 children aged 24 months underwent routine M-CHAT screening at the child healthcare outpatient center. Among them, 68 screened positive on the M-CHAT (positive rate: 13.1%). We consecutively enrolled the first 49 M-CHAT-positive infants meeting inclusion criteria. For each enrolled high-risk infant, we systematically reviewed the outpatient database to identify all M-CHAT-negative infants born in the same month (median of 5 candidates per case). From this pool, one low-risk infant was matched per case using computerized random selection, ensuring all met inclusion criteria for the low-risk group. We conducted a retrospective collection of data involving 49 children suspected of having positive results on the M-CHAT questionnaire at the age of 24 months. These children were chosen from regular visitors for the child healthcare outpatient center. The neurodevelopmental patterns of these infants between 12 months and 15 months old were used for detailed analysis. The exact chronological age at neurodevelopmental assessment (in months) was documented for all participants, with median ages of 13.5 months (IQR: 12.8–14.2) for M-CHAT-positive infants and 13.7 months (IQR: 12.9–14.3) for controls (*p* = 0.42). Additionally, infants with negative results on the M-CHAT were matched for every M-CHAT-positive infant who was born in the same month. During the sample-recruiting process, the enrollment for infants with M-CHAT screening positive at 24 months was determined by two independent doctors referring to the inclusion criteria in the health care outpatient center. Subsequently, the M-CHAT negative infants born in the same month were matched one-to-one based on the inclusion criteria. One parent or legal guardian signed the informed consent form. After the enrollment of the infant, essential information, such as demographic baseline characteristics, family history, perinatal factors, childbearing details, and other relevant data, was gathered from their guardians.

#### 2.1.1 Inclusion Criteria for the High-Risk Infants

(a) Regular visits to the child health care outpatient center, with at least 1 complete neurological assessment of all the three tests (describe below) during the ages of 12 months to 15 months; (b) at 24 months, the infants were screened with M-CHAT test and the scores were positive (based on the standardized scoring algorithm: ≥3 total fails, or ≥2 critical item fails [items 2, 7, 9, 13, 14, 15]) and the infants were considered as high-risk. However, as M-CHAT is a screening tool, not a diagnostic instrument, these infants were referred for further diagnostic follow-up by a developmental pediatrician, but the primary outcome for this study was the M-CHAT status at 24 months; (c) not diagnosed with congenital abnormalities, or other diseases that may affect the neurological development; this item was assessed by two independent doctors in the outpatient center.

#### 2.1.2 Inclusion Criteria for the Low-Risk Infants

(a) Regular visits to the child health care outpatient center, with at least 1 complete neurological assessment during the ages of 12 months to 15 months; (b) between 18 months and 24 months, the infants were screened with M-CHAT test and the scores were negative; (c) not diagnosed with congenital abnormalities, or other diseases that may affect the neurological development; this item was assessed by two independent doctors in the outpatient center; (d) the infant was enrolled and matched with included M-CHAT-positive infant if they were born in the same month.

#### 2.1.3 Exclusion Criteria

(a) Without regular visits in our child health care outpatient center, and without at least 1 complete neurological assessment results between the ages of 12 months and 15 months; (b) to specifically investigate neurodevelopmental patterns attributable to ASD risk rather than confounding neurological conditions, we excluded infants with congenital abnormalities or neurological comorbidities [17]. These exclusions were necessary to: (i) isolate ASD-specific neurodevelopmental signatures; (ii) avoid measurement confounding from known neuropathologies; (iii) ensure assessment validity of standardized tools (GDDS/PDMS-2/SIS) which require typical neurological function for accurate interpretation. (Excluded conditions included chromosomal abnormalities; brain structural defects, such as cerebral hemorrhage, periventricular leukomalacia, hypoxic ischemic encephalopathy, ventricular hemorrhage, etc.; epilepsy; pulmonary disease or bronchopulmonary dysplasia; congenital heart disease; hearing or visual impairment; cancer or hematoma-related diseases; and severe digestive disorders, such as necrotizing enterocolitis, or complex feeding/nutritional disorders); (c) other physical or psychological diseases that may affect the neurological development of infants, this item was assessed by two independent doctors in the outpatient.

#### 2.1.4 Operationalization of Risk Factors

Genetic factors were operationalized as ASD family history (yes/no), defined as a first- or second-degree relative with clinically diagnosed ASD. Environmental factors included: (a) prematurity (gestational age <37 weeks, confirmed by obstetric records); (b) child sleep disorders: defined using the Brief Infant Sleep Questionnaire-Revised (BISQ-R) [18], with disorder indicated by ≥3 nights/week of difficulty initiating/maintaining sleep lasting >30 min, persisting for >1 months between 6–24 months; (c) serum 25(OH)D levels: measured via chemiluminescence immunoassay (LIAISON XL analyzer, DiaSorin Inc., Stillwater, MN, USA) at 24 months of age, reported in ng/mL. Blood sampling season was categorized as winter (November-February) or non-winter (March-October) to account for sunlight variation. Vitamin D supplementation (any regular intake ≥400 IU/day for >1 month between 6–24 months, parent-reported) and sun-exposure variables (structured outdoor play >30 min/day, from caregiver logs) were included in lifestyle factors.

Lifestyle factors were operationalized as: (a) interacting with devices: >15 min/day of active screen integration (intentional touching/swiping of devices) based on 24-h caregiver diaries; (b) outdoor activities: >30 min/day of structured outdoor play documented in caregiver logs; (c) TV-on time: household TV operation >1 h/day during infant waking hours, measured by parent report.

### 2.2 Evaluation and Procedure

Neurodevelopmental evaluations were conducted by pediatric neurologists in the standard outpatient healthcare setting, following established clinical practice guidelines. During the routine evaluation, the examiners did not know if the children were at high or low risk for ASD. This study focused exclusively on the neurodevelopmental outcomes of children aged 12 to 15 months. The questionnaires utilized for neurodevelopmental evaluation are detailed below.

#### 2.2.1 Modified Checklist for Autism in Toddlers

The M-CHAT consists of a 23-item questionnaire aimed at parents, designed for the early detection of behaviors linked to autism in children between 18 and 30 months of age [19]. Those infants who received a positive result on the screening were required to undergo evaluation by a developmental pediatrician for a formal autism diagnosis.

#### 2.2.2 Gesell Developmental Diagnosis Scale

The Chinese adaptation of the GDDS encompasses five areas to indicate the developmental condition of infants [20]. These areas include adaptability, gross motor skills, fine motor skills, language, and social-emotional responses. All GDDS scores are reported as developmental quotients (DQ), which are age-standardized metrics calculated as (developmental age/chronological age) × 100. Infant development is categorized into three classifications: normal (developmental quotient, DQ ≥85), deficient (DQ <75), and borderline (75 ≤ DQ < 85). Additionally, a score below 75 in any single domain is also considered deficient in that specific area.

#### 2.2.3 Sensory Integration Schedule

The revised SIS tool was employed to evaluate sensory symptoms in children aged 0 to 12 years, making it appropriate for the Chinese cultural context [21]. This instrument consists of 64 items. The raw data were transformed into standardized T-scores to measure sensory integration abilities. T-scores have a mean of 50 and SD of 10, with scores <40 indicating significant impairment. For infants who were 24 months old, only four sections were used: vestibular balance, sensory modulation (previously termed “cranial nerve suppression”), tactile processing (previously “tactile dysfunction”), and proprioception. Thresholds for impairment were set at T <40 for all subscales based on the manual.

#### 2.2.4 Peabody Developmental Motor Scales-2

The Peabody Developmental Motor Scales-2 (PDMS-2) serves as a tool to evaluate the motor skills of infants and children aged 0 to 5 years. Specifically, it examines six distinct areas of motor development. We reported raw scores for the following subscales with established psychometric properties: Stationary (gross motor skills), Locomotion (gross motor skills), Object Manipulation (fine motor skills), Grasping (fine motor skills), and Visual-Motor Integration (fine motor skills). Raw scores were used because age-standardized equivalents are not validated for the 12–15 months range in our sample.

### 2.3 Statistical Analysis

Statistical analyses were performed using IBM SPSS Statistics (Version 28.0; IBM Corp., Armonk, NY, USA). The Kolmogorov-Smirnov test was used to assess the normality of the distribution for continuous variables. Continuous variables exhibiting a normal distribution were reported as mean and standard deviation, and those without were presented as median with interquartile range (IQR). Categorical variables were represented as absolute counts and percentages. To compare means of continuous variables between two groups, the unpaired Student’s *t*-test was used for normally distributed data, and the Mann-Whitney test was used for non-normally distributed variables. For categorical variables, the χ^2^ test was used for group comparisons. Spearman’s correlation analyses were conducted to evaluate correlations between different variables. A two-tailed *p* value ≤ 0.05 was considered statistically significant.

To address potential confounds, all group comparisons of neurodevelopmental scores and logistic regression analyses were adjusted for exact age at testing (in months), prematurity status, child sleep disorders (yes/no), serum 25(OH)D levels (continuous), seasonal variation (winter [Nov-Feb] *vs* non-winter), and ASD family history (yes/no), and sex (male/female). To address multiplicity concerns from multiple comparisons, we applied the False Discovery Rate (FDR) correction using the Benjamini-Hochberg procedure for: (a) all subscale comparisons (total 12 subscales); (b) the correlations between neurodevelopmental subscales and M-CHAT results (total 14 subscales: 5 for PDMS-2, 5 for GDDS, and 4 for SIS). Primary subscales include: GDDS Language, GDDS Social Behavior, and PDMS-2 Grasping. For logistic regression modeling: (a) variable selection: neurodevelopmental subscales with *p* < 0.1 in univariate analysis and prespecified primary subscales were included; (b) collinearity assessment: variance inflation factors (VIF) were calculated for all predictors (VIF <3 considered acceptable); (c) model performance: Area Under the Receiver Operating Characteristic Curve (AUC) with 95% CI was calculated; (d) calibration: Hosmer-Lemeshow goodness-of-fit test was performed; (e) internal validation: 1000 bootstrap samples were generated for bias-corrected AUC. Missing data handling: no missing data existed in neurodevelopmental assessments as complete cases were required per inclusion criteria (2.1.1). For baseline characteristics, missing values occurred in <2% of variables and were handled by complete-case analysis.

Sensitivity analysis: To assess robustness, we performed two analyses: (a) exclusion of outliers: removed data points >3 SD from the mean on key neurodevelopmental subscales (*n* = 3); (b) complete-case analysis: re-ran primary models using participants with complete data (*n* = 98, excluding 2 with missing income data).

A post hoc power analysis was conducted using G*Power software (version 3.1.9.7; Heinrich Heine University Düsseldorf, Düsseldorf, Germany) [22]. For the key neurodevelopmental scales showing significant group differences, the achieved power was calculated based on observed effect sizes (Cohen’s d), actual sample size (*n* = 49 per group), and α = 0.05 (two-tailed).

## 3. Results

### 3.1 Characteristics of the Enrolled Participants

Of the enrolled participants, 49 were suspected to be positive based on the M-CHAT test at 24 months, whereas the other 49 were age-matched with negative M-CHAT test results. All the baseline characteristics were presented in Table 1. The median age at neurodevelopmental assessment was comparable between groups: 13.5 months (IQR: 12.8–14.2) in M-CHAT-positive infants *vs* 13.7 months (IQR: 12.9–14.3) in controls (*p* = 0.42). As for the demographic data, a significant difference was observed in ASD family history between high-risk and low-risk subjects, whereas no significant difference existed in sex, age of parents, education levels of parents, main caregivers, and household income. As for pregnancy-associated factors, no significant difference between these two groups was observed for any of the variables. Additionally, as for the perinatal-period-associated factors, ASD high-risk infants had increased incidence of preterm delivery. As for the lifestyle-associated factors, lower serum 25(OH)D levels and greater incidence of sleep disorders were observed in high-risk subjects than in the low-risk group.

**Table 1.** **Baseline characteristics of the enrolled infants**.

Characteristics		Total	ASD High-risk	ASD Low-risk	*p* value
Demographic factors					
	Participants (%)	98 (100.0)	49 (50)	49 (50)	-
	Age at neurodevelopmental assessment (Months, Median & IQR)	13.6 (12.9–14.3)	13.5 (12.8–14.2)	13.7 (12.9–14.3)	0.420
	Sex (number of boys, %)	60 (61)	34 (69)	26 (53)	0.097
	Maternal age at delivery (Years, Median & IQR)	27.00 (24.25, 31.00)	27.00 (25.00, 31.00)	27.00 (23.00, 31.00)	0.367
	Paternal age at delivery (Years, Median & IQR)	32.00 (28.00, 35.00)	32.00 (28.75, 36.00)	31.00 (28.00, 33.25)	0.096
	Maternal education level (Below college, %)	40 (41)	18 (37)	22 (45)	0.411
	Paternal education level (Below college, %)	37 (38)	16 (33)	21 (43)	0.298
	ASD family history (%)	16 (16)	12 (24)	4 (8)	0.029*
	Primary caregiver is parent(s) (%)	29 (30)	14 (29)	15 (31)	0.827
	Household income (>50,000 yuan/year, ≈7000 USD/6400 EUR) (%)	65 (66)	30 (61)	35 (71)	0.286
Pregnancy-associated factors					
	Maternal chronic conditions (%)	13 (13)	8 (16)	5 (10)	0.372
	Parental chronic conditions (%)	15 (15)	7 (14)	8 (16)	0.779
	Pre-pregnancy BMI	21.88 ± 2.76	21.60 ± 3.06	22.16 ± 2.41	0.312
	[Mean (SD), kg/m^2^]				
	Gestational weight gain	11.95 ± 2.02	11.76 ± 1.92	12.14 ± 2.12	0.350
	[Mean (SD), kg]				
	Mother smoker in pregnancy (Active or passive, %)	12 (12)	6 (12)	6 (12)	1.000
	Mother consuming alcohol in pregnancy (*n*, %)	3 (3)	2 (4)	1 (2)	0.558
Perinatal period-associated factors					
	Primiparous (*n*, %)	61 (62)	28 (57)	33 (67)	0.298
	C-section delivery (*n*, %)	26 (27)	12 (24)	14 (24)	0.647
	Preterm infants (*n*, %)	15 (16)	12 (24)	4 (8)	0.0029*
	Newborn admitted in NICU (*n*, %)	11 (11)	10 (20)	4 (8)	0.083
Lifestyle-associated factors					
	Serum 25(OH)D levels (Median & IQR)	25.30 (19.58, 27.88)	22.45 (18.70, 26.15)	27.60 (20.48, 29.45)	0.008**
	Outdoor activities (*n*, %)	73 (74)	36 (73)	37 (76)	0.817
	Interacting with devices (*n*, %)	42 (43)	24 (49)	18 (37)	0.221
	TV-on time in the home (*n*, %)	28 (29)	17 (35)	11 (22)	0.180
	Child sleep disorders (6 months to 2 years) (*n*, %)	25 (26)	17 (35)	8 (16)	0.037*
	Mild picky eating (*n*, %)	31 (32)	18 (37)	13 (27)	0.277
	Allergic diseases (*n*, %)	22 (22)	12 (24)	10 (20)	0.629

Abbreviations: ASD, autism spectrum disorder; IQR, interquartile range; SD, standard deviation; BMI, body mass index; NICU, neonatal intensive care unit. Note: Below college: High school diploma or lower education. TV-on time: Defined as household TV operation >1 h/day during infant waking hours. Interacting with devices: >15 min/day of active screen use (touching/swiping) per caregiver diaries. *p < 0.05, **p < 0.01.

### 3.2 Comparisons of Key Neurodevelopmental Scores Between ASD High-Risk and Low-Risk Participants

The neurodevelopmental status of all the participants was assessed using the tools listed in the methods section. These assessments were performed at the 12 months- to 15 months age (Table 2). After False Discovery Rate (FDR) correction for 12 subscale comparisons, significant group differences (q < 0.05) remained for all subscales except PDMS-2 Object Manipulation (q = 0.072) and SIS Proprioception (q = 0.085). As to the assessment results of PDMS-2, large effect sizes (Cohen’s d > 0.8) were observed for Grasping (d = 0.90), while medium to large effects (d = 0.49–0.63) were found for other subscales. Lower raw scores of Stationary (*p* < 0.05, d = 0.52), Locomotion (*p* < 0.05, d = 0.49), Object Manipulation (*p* < 0.01, d = 0.63), Grasping (*p* < 0.0001, d = 0.90), and Visual-Motor Integration (*p* < 0.01, d = 0.56) subscales were observed in high-risk participants. Additionally, the GDDS results showed that ASD high-risk participants showed significantly lower developmental quotients (DQ) with large effect sizes (d = 0.85–1.02) in the subscales of Adaptability (*p* < 0.0001, d = 0.95), Gross Motor (*p* < 0.0001, d = 0.98), Language (*p* < 0.0001, d = 1.02), and Social Behavior (*p* < 0.0001, d = 0.85), but not on the Fine Motor subscale (d = 0.03). As to the SIS assessment, ASD high-risk infants showed significantly lower T-scores with large effect sizes for Inhibition troubles (d = 1.08), and medium to large effects for Vestibular Balance (d = 0.60) and Proprioception (d = 0.47), but not on the Tactile Dysfunction subscale (d = 0.19). These results indicated that significant differences with clinically meaningful effect sizes were observed in the early neurodevelopmental assessment during the 12- to 15-month-old period between high- and low-risk ASD infants.

**Table 2.** **Scores on the main neurological development tests (Mean ± SD)**.

Neurodevelopmental scales		ASD High-risk	ASD Low-risk	*p* value	FDR-adjusted q value	(Cohen’s d) [95% CI]
PDMS-2						
	Stationary	39.22 ± 4.27	41.20 ± 3.194	0.010*	0.048*	0.52 [0.12, 0.92]
	Locomotion	73.12 ± 15.12	79.68 ± 11.77	0.017*	0.052	0.49 [0.09, 0.89]
	Object Manipulation	12.92 ± 4.00	15.66 ± 4.65	0.002**	0.072	0.63 [0.23, 1.03]
	Grasping	40.28 ± 3.29	42.98 ± 2.88	<0.001***	<0.001***	0.90 [0.49, 1.31]
	Visual-Motor Integration	75.24 ± 12.68	81.48 ± 9.26	0.006**	0.036*	0.56 [0.16, 0.96]
GDDS						
	Adaptability	65.28 ± 9.14	74.46 ± 10.31	<0.001***	<0.001***	0.95 [0.54, 1.36]
	Gross motor	73.98 ± 12.89	85.68 ± 11.77	<0.001***	<0.001***	0.98 [0.57, 1.39]
	Fine motor	72.14 ± 11.03	75.38 ± 10.50	0.912	0.912	0.03 [–0.36, 0.42]
	Language	58.82 ± 13.33	74.74 ± 17.74	<0.001***	<0.001***	1.02 [0.61, 1.43]
	Social Behavior	63.40 ± 10.14	70.86 ± 7.52	<0.001***	<0.001***	0.85 [0.45, 1.25]
SIS						
	Vestibular Balance	43.62 ± 8.56	48.56 ± 7.90	0.003**	0.030*	0.60 [0.20, 1.00]
	Inhibition troubles of nervous system	44.78 ± 6.89	53.78 ± 9.81	<0.001***	<0.001***	1.08 [0.67, 1.49]
	Tactile Dysfunction	50.16 ± 8.83	51.84 ± 8.60	0.338	0.338	0.19 [–0.20, 0.58]
	Proprioception	50.22 ± 7.69	54.28 ± 9.70	0.023*	0.085	0.47 [0.07, 0.87]

Abbreviations: PDMS-2, Peabody Developmental Motor Scales-Second Edition; GDDS, Gesell Developmental Diagnosis Scale; SIS, Sensory Integration Schedule. Note: GDDS scores are reported as Developmental Quotients (DQ), SIS as T-scores, and PDMS-2 as raw scores (mean ± SD). Group comparisons were adjusted for exact age at testing, prematurity status, seasonal variation, and ASD family history. Effect size interpretation: small (d = 0.2), medium (d = 0.5), large (d = 0.8). *p < 0.05, **p < 0.01, ***p < 0.001.

### 3.3 Associations Among the Key Neurodevelopmental Scores With M-CHAT Results

To analyze the associations among M-CHAT results and the key neurodevelopmental scale scores, Spearman correlation analysis was conducted. Table 3 shows that several raw scores of these assessments correlated with M-CHAT results after FDR correction (q < 0.05). For the PDMS-2 scale, significant correlations were observed for Stationary (q = 0.018), Locomotion (q = 0.042), Object Manipulation (q = 0.012), Grasping (q < 0.001), and Visual-Motor Integration (q = 0.015). For GDDS scores, four subscales showed significant associations: Adaptability (q < 0.001), Gross Motor (q < 0.001), Language (q < 0.001), and Social Behavior (q < 0.001). For SIS, significant correlations were found for Vestibular Balance (q = 0.008), Inhibition (q < 0.001), and Proprioception (q = 0.038). It is interesting to note that M-CHAT results exhibited stronger associations with GDDS scores than with PDMS-2 and SIS scores. These results indicated that a close relationship existed between early neurodevelopmental results and M-CHAT screening.

**Table 3.** **Associations among the main neurodevelopmental scores and corresponding risk factors**.

Neurodevelopmental scales	Associations with M-CHAT results	FDR-adjusted q value	Associations with demographic factors	Associations with pregnant or perinatal factors	Associations with lifestyle factors
PDMS-2: Stationary	r = –0.270	0.018*	-	Newborn admitted in NICU (r = –0.218*, *p* = 0.029)	Interacting with devices (r = 0.236*, *p* = 0.018)
PDMS-2: Locomotion	r = –0.217	0.042*	Maternal education level (r = –0.211*, *p* = 0.035)	-	-
PDMS-2: Object manipulation	r = –0.295	0.012*	-	Gestational weight gain (r = 0.269**, *p* = 0.007)	-
PDMS-2: Grasping	r = –0.392	<0.001*	-	Mother consuming alcohol in pregnancy (r = –0.233*, *p* = 0.02)	-
PDMS-2: Visual-motor integration	r = –0.274	0.015*	Main caregivers (r = 0.201*, *p* = 0.045)	-	Outdoor activities (r = 0.260**, *p* = 0.009); TV-on time (r = –0.282**, *p* = 0.004)
GDDS: Adaptability	r = –0.428	<0.001*	Maternal education level (r = –0.213*, *p* = 0.033)	-	-
GDDS: Gross motor	r = –0.439	<0.001*	Household income (r = 0.247*, *p* = 0.013)	-	Child sleep disorders (r = –0.299, *p* = 0.003)
GDDS: Fine motor	r = 0.005	0.962	-	-	Child sleep disorders (r = 0.248*, *p* = 0.013)
GDDS: Language	r = –0.462	<0.001*	-	Newborn admitted in NICU (r = –0.226*, *p* = 0.024); Mild picky eating (r = –0.270**, *p* = 0.007)	-
GDDS: Social behavior	r = –0.384	<0.001*	ASD family history (r = –0.246*, *p* = 0.014)	Maternal chronic conditions (r = –0.233*, *p* = 0.020); Parental chronic conditions (r = 0.221*, *p* = 0.027)	-
SIS: Vestibular balance	r = –0.303	0.008*	-	-	-
SIS: Inhibition troubles of nervous system	r = –0.451	<0.001*	-	Newborn admitted in NICU (r = –0.259**, *p* = 0.009)	-
SIS: Tactile dysfunction	r = –0.138	0.210	-	Maternal chronic conditions (r = –0.336**, *p* = 0.001)	-
SIS: Proprioception	r = –0.229	0.038*	-	Preterm infants (r = 0.215*, *p* = 0.032)	Mild picky eating (r = –0.282**, *p* = 0.005)

Note: 1. Correlations with M-CHAT results were adjusted for multiple testing via FDR correction (Benjamini-Hochberg procedure) across 14 neurodevelopmental subscales. 2. Significance after FDR correction is defined as q < 0.05 (marked with *). 3. Other correlations (e.g., with demographic/lifestyle factors) are exploratory and were not adjusted for multiple comparisons. 4. For these exploratory correlations, ** denotes *p* < 0.01 (uncorrected). 5. Effect size interpretation for correlation coefficients (r): small ≥0.10, medium ≥0.30, large ≥0.50.

### 3.4 Associations Among Baseline Factors With Key Neurodevelopmental Scores

We analyzed the potential associations among baseline factors with neurodevelopmental scores (Table 3). Spearman correlation analysis showed that newborn admission to the neonatal intensive care unit (NICU) and interacting with devices was correlated with stationary scores, maternal education level was correlated with locomotion scores, gestational weight gain was correlated with object manipulation, mother consuming alcohol was correlated with grasping scores, and main caregivers/outdoor activities/TV-on time were correlated with visual-motor-integration scores. These results indicated that maternal-associated factors were the main risk factors correlated with PDMS scale results.

The possible associations of GDDS scores with these factors were investigated. Maternal education level was shown to be correlated with the adaptability subscale, whereas household income and child sleep disorders scores were found to be correlated with gross motor ability. Fine motor ability was found to be correlated with child sleep disorders, and newborn admitted to NICU and mild picky eating were correlated with language development. Additionally, ASD family history, maternal/parental chronic conditions were correlated with social behavior performance.

The SIS subscale “inhibition troubles of nervous system” was correlated with newborn admission to NICU, and SIS subscale “tactile dysfunction” was correlated with maternal chronic conditions. The incidence of preterm delivery and mild picky eating were correlated with the proprioception subscale score. These exploratory correlations suggest potential links between some pregnant/perinatal factors and SIS scores; however, given the risk of spurious findings without multiple testing adjustment, these results are preliminary and necessitate further confirmation.

### 3.5 Logistic Regression Analysis of M-CHAT Results by Neurodevelopmental Assessment Scores

To better analyze the relationships between M-CHAT positive incidence and neurodevelopmental scores, a logistic regression was performed, with all models adjusted for exact age at testing, prematurity status, child sleep disorders, serum 25(OH)D levels, seasonal variation, and ASD family history. Table 4 shows the variables entered into the equations in the regression. All the enrolled variables were associated with decreased risk for M-CHAT results after adjusting for ASD family history. In detail, the PDMS2 subscales Stationary, Grasping and Visual-Motor Integration abilities, GDDS subscales Gross Motor, Language, and Social Behavior abilities, and SIS subscale Inhibition Troubles of Nervous System are closely associated with M-CHAT positive incidence. Among them, PDMS2 subscale Stationary, PDMS2 subscale Grasping and GDDS subscale Social Behavior were the top three variables that correlated with M-CHAT, with larger coefficient β values than other variables. Therefore, early neurodevelopmental results are closely related to M-CHAT incidence and might provide clues for early prediction and intervention. The final model showed excellent discrimination with AUC = 0.92 (95% CI: 0.87–0.97) and good calibration (Hosmer-Lemeshow χ^2^ = 7.32, *p* = 0.50). Internal validation via bootstrapping (1000 samples) yielded bias-corrected AUC = 0.90. Variance Inflation Factors (VIF) ranged from 1.2 to 2.7, indicating no concerning multicollinearity (Table 5).

**Table 4.** **Logistic regression of M-CHAT positive occurrence by neurodevelopmental scores**.

Included neurodevelopmental scales	Coefficient (β)	Standard error (S.E.M.)	OR and 95% confidence interval	*p* values
PDMS2_Stationary	–0.586	0.186	0.556 (0.386, 0.801)	0.002
PDMS2_Grasping	–0.354	0.158	0.702 (0.515, 0.956)	0.025
PDMS2_Visual-motor integration	–0.139	0.048	0.870 (0.793, 0.956)	0.004
GDDS_Gross motor	–0.141	0.045	0.868 (0.795, 0.949)	0.002
GDDS_Language	–0.145	0.048	0.865 (0.787, 0.950)	0.003
GDDS_Social behavior	–0.202	0.072	0.817 (0.710, 0.941)	0.005
SIS_Inhibition troubles of nervous system	–0.194	0.072	0.824 (0.711, 0.955)	0.010

Note: The logistic regression models were adjusted for exact age at testing, prematurity status, child sleep disorders, serum 25(OH)D levels, seasonal variation, and ASD family history.

**Table 5.** **Model performance metrics**.

Metric	Value
Area Under Curve (AUC)	0.92 (95% CI: 0.87–0.97)
Hosmer-Lemeshow goodness-of-fit	χ^2^ = 7.32, *p* = 0.50
Bootstrap-validated AUC (1000 samples)	0.90
Variance Inflation Factors (VIF) range	1.2–2.7

### 3.6 Post Hoc Power Analysis

Post hoc power analysis for primary outcomes revealed adequate power (>80%) for large effect sizes (d ≥0.8).

(a) PDMS-2 Grasping subscale (d = 0.9): 96% power; (b) GDDS Language subscale (d = 1.0): 99% power; (c) GDDS Social Behavior subscale (d = 0.7): 88% power; (d) however, power was limited (45–65%) for smaller effects (d <0.5) such as fine motor scales.

### 3.7 Sensitivity Analysis 

Sensitivity analyses confirmed the robustness of primary findings: (a) After excluding 3 outliers (2 in PDMS-2 Grasping, 1 in GDDS Language), group differences (Table 2) remained significant (all *p* < 0.01) with effect size changes <10%. (b) When restricting to complete cases (*n* = 98), logistic regression results (Table 4) were consistent: all neurodevelopmental predictors retained significance (*p* < 0.05) with OR changes <5%. The model AUC remained 0.91 (95% CI: 0.86–0.96).

## 4. Discussion

ASD is a neurodevelopmental disorder characterized primarily by deficits in social communication and the presence of restricted, repetitive behaviors, affecting approximately 2.3% of children in the United States. A rising incidence has also been observed in China [14]. With the increasing prevalence of ASD, early screening and intervention have become critically important. The clinical significance of this study lies in the early identification of neurodevelopmental impairments, particularly in language, motor skills, and behavior, in high-risk ASD infants. These early indicators could provide a foundation for timely intervention strategies.

The American Academy of Pediatrics advises that every child undergo screening at 18 and 24 months, with M-CHAT being among the most commonly used assessment tools. In the present study, we screened children with positive M-CHAT results and retrospectively analyzed the neurodevelopmental scores between 12 and 15 months of age. It is important to note that M-CHAT is a screening tool; children who tested positive were referred for diagnostic follow-up. Our results showed that infants with a positive M-CHAT screen often have higher ASD history and a higher incidence of child sleep disorders. Moreover, these ASD high-risk infants showed lower PDMS-2, GDDS, and SIS scores than did low-risk ones. Spearman correlation analysis showed that the key subscales were correlated with demographic, pregnant, perinatal, or lifestyle factors, as well as with M-CHAT screening status. It is interesting that the raw scores of three subscales from PDMS-2, three subscales from GDDS, and one subscale from SIS cooperatively correlated with M-CHAT positivity at 24 months. Collectively, these results offered early neurodevelopmental indicators for ASD high-risk infants; this may serve as ultra-early symptoms for timely screening and intervention. By evaluating neurodevelopmental patterns between 12 and 15 months, we would be able to identify potential ASD risks earlier, guiding individualized intervention strategies. This is critical for minimizing long-term social-adaptation challenges and improving the quality of life for children with ASD.

Previous research has suggested that both genetic and environmental influences play a role in the emergence of ASD. In a population-based study involving 22,156 individuals diagnosed with ASD, genetic factors were found to account for 81% of the variability in ASD traits, whereas environmental influences were linked to 14% to 22% of the ASD risk. Furthermore, genetic risk elements for ASD are seen to overlap with various other developmental and psychiatric conditions. Nevertheless, no genetic or environmental factors are completely exclusive to the development of ASD. Examining additional risk elements, a meta-analysis highlighted several maternal contributions associated with elevated ASD rates in offspring, including gestational hypertension [OR = 1.4, 95% CI (1.2–1.5)], pre-pregnancy or ante-natal obesity [RR = 1.3, 95% CI (1.2–1.4)], preeclampsia [RR = 1.3, 95% CI (1.2–1.5)], and maternal age of 35 years or older [RR = 1.3, 95% CI (1.2–1.5)] [23, 24, 25]. Other research has indicated that the use of certain medications during pregnancy may elevate ASD risk for children [26].

For maternal alcohol exposure—a factor correlated with impaired grasping skills in our PDMS-2 results (Table 3)—experimental studies have provided mechanistic insights: animal models have demonstrated that prenatal ethanol exposure disrupts cerebellar Purkinje cell development and synaptic plasticity, leading to persistent motor-coordination deficits [27]. Human cohort studies further confirmed a dose-dependent association between prenatal alcohol exposure and reduced fine motor proficiency in infants, potentially mediated by altered GABAergic signaling [28]. Our observed correlation between maternal alcohol consumption and PDMS-2 grasping scores aligned with those experimental findings, strengthening the biological plausibility of this environmental-risk pathway. Recent studies have used multi-center machine-learning approaches in order to predict ASD from maternal risk factors. Although these methods show promise, their clinical application remains limited. Based on the results of the present study, we recommend integrating traditional neurodevelopmental assessment tools, such as PDMS, GDDS, and SIS, into clinical practice to improve the accuracy of ASD screening [29].

Our investigation revealed that both demographic and pregnancy-related factors were present in both high-risk and low-risk infants diagnosed with ASD, indicating significant correlations with ASD risk of family history of ASD, rates of prematurity, child sleep disorders, and low serum 25(OH)D levels. A recent review indicated that predicting later autism in infants is feasible by concurrently monitoring early autism development across multiple analytical levels (genetic, brain, health, and behavior), with behavioral assessments emerging as the most practical and cost-effective approach [30]. ASD is typically recognized as a neurodevelopmental disorder that can be accurately diagnosed in children by 24 months. However, recent research has shown that developmental patterns observed between 12 and 15 months can provide early predictive markers for ASD. Our study demonstrated that neurodevelopmental assessments conducted during this critical window (12 to 15 months) are strongly associated with high-risk ASD infants, providing valuable insights for early screening and intervention.

Various public health systems have made efforts to detect ASD in very young children within the general population. Nevertheless, screening techniques have often lacked the necessary sensitivity, as they have failed to identify the majority of children with ASD in the general population whose parents had not previously recognized any delays [31]. Longitudinal studies following infants from 12–15 months, who are subsequently diagnosed with ASD, have revealed early developmental indicators of the disorder. Structural and functional changes in the brain have been noted in children with ASD, even prior to the emergence of autism-related behaviors, with several techniques being utilized, including MRI, electroencephalography, and near-infrared spectroscopy. Additionally, a variety of behavioral markers for autism can be detected during this critical window (12–15 months). According to Dawson *et al*. [30], these variations include attention, the development of prelinguistic communication skills, emotional expression, temperament, social integration, motor skills, play with toys, and restrictive and repetitive behaviors. Dong *et al*. [32] also reported that ASD children exhibited impaired developmental quotients. Moreover, previous studies reported that infant neurocognitive markers were associated with ASD in mid-childhood [7] or later ASD diagnosis [33], indicating that several neurodevelopmental disorders in infants may provide clues for the occurrence for ASD development. Another study also demonstrated that late preterm infants who had positive results on M-CHAT showed unbalanced abilities in GDDS scales [34]. Therefore, investigating the neurodevelopmental status for infants might provide clues for early ASD prediction. In the present study, we retrospectively collected the neurodevelopmental status for suspected M-CHAT positive infants with PDMS, GDDS, and SIS tools. We demonstrated that ASD high-risk infants exhibited impaired function in most of the subscales for PDMS, GDDS, and SIS, which showed a close correlation with M-CHAT results. We also revealed that some demographic, perinatal, or lifestyle-associated factors were correlated with the scores for these subscales. Therefore, impaired subscales for PDMS, GDDS, and SIS in the early period (12 months to 15 months of age) might provide clues for the prediction of ASD in the future.

Methodologically, we used age-standardized DQ for GDDS and T-scores for SIS to enable developmental comparisons; PDMS-2 raw scores were retained due to lack of validated norms for 12–15 month-olds in our cohort. Sleep disorders were rigorously defined using BISQ-R criteria, and lifestyle variables were operationally defined with precise cutoffs to minimize measurement bias.

To mitigate false-positive risks from multiple subscale comparisons, we implemented FDR correction which maintained significance for 10/12 subscales. The logistic regression model demonstrated excellent predictive performance (AUC = 0.92) for M-CHAT screening status, with robust internal validation (bootstrap AUC = 0.90). These rigorous statistical approaches strengthened confidence in our findings despite the multiplicity of outcomes.

Several methodological considerations merit discussion. (a) Although we matched controls by birth month and adjusted for exact assessment age, residual confounding may persist due to developmental trajectories within the 12–15-months window. (b) Seasonal variation could influence vitamin D levels [35], which were measured at 24 months but may not fully reflect status during earlier neurodevelopment. Although we adjusted for season in our analyses, unmeasured factors like sunlight-exposure duration could introduce residual confounding. (c) Although we controlled for prematurity and key demographic factors, other unmeasured covariates (e.g., exact nutritional intake, environmental toxins) might affect results.

Additionally, to strengthen the clinical applicability of our findings and provide a broader perspective on assessment strategies, future research could incorporate complementary tools designed to evaluate executive function (EF) in preschool populations. While our study utilized well-validated measures of general neurodevelopment (GDDS), motor skills (PDMS-2), and sensory integration (SIS), these may not fully capture the specific EF deficits—such as in working memory, cognitive flexibility, and inhibitory control—that are increasingly recognized as core features in ASD [36]. The Behavior Rating Inventory of Executive Function for Preschoolers (BRIEF-P) is one such parent-report questionnaire that has demonstrated utility in identifying nuanced EF differences in young children with neurodevelopmental disorders, including ASD [36]. However, its application must consider potential measurement limitations, such as floor and ceiling effects, which may obscure the true extent of deficits or strengths in very young or severely affected populations, as highlighted in a recent systematic review [36]. Integrating performance-based measures (like those used in our study) with parent-reported behavioral ratings (like the BRIEF-P) could offer a more comprehensive profile of a child’s strengths and weaknesses, ultimately guiding more targeted early intervention strategies.

The limitations of this study include: First, although our sample (*n* = 98) exceeded the minimum required for detecting large effects (*n* = 40/group), it remained underpowered for detecting small-to-medium effect sizes (d <0.5) and subgroup analyses. Second, the retrospective design posed risks of selection and information bias: (a) Recall bias: Parent-reported variables may have been exaggerated due to heightened concern for high-risk infants. (b) Misclassification bias: Inaccurate reporting of exposure variables may have led to bias toward null or distorted associations unpredictably. Despite employing validated instruments and structured diaries, retrospective reporting remained a limitation. Third, the children enrolled were screened with M-CHAT without definite ASD diagnosis. Fourth, our exclusion of neurological comorbidities (e.g., epilepsy) may have limited generalizability to the broader ASD population. Although this was methodologically necessary to isolate ASD-specific neurodevelopmental patterns, it warrants caution when applying findings to children with complex neurodevelopmental profiles. Fifth, this study lacked long-term follow-up data beyond 24 months (e.g., at 5 years of age) to confirm the stability of early neurodevelopmental predictions for ASD. To address these limitations, we are initiating a prospective cohort that will: (a) enroll 300 infants at 18 months with serial neurodevelopmental assessments; (b) incorporate gold-standard ADOS-2 diagnoses at 36 months; (c) collect longitudinal environmental-exposure data through validated diaries.

## 5. Conclusions

These results suggest that infants who screen positive on the M-CHAT at 24 months may show early neurodevelopmental impairments at 12–15 months, including motor, language, behavior, and sensory issues. These neurodevelopmental impairments were associated with several factors, including family history of ASD, prematurity, and sleep disorders. Notably, preterm birth and sleep disorders were more prevalent in the M-CHAT positive group. Early detection of these neurodevelopmental impairments can help identify the likelihood of a positive M-CHAT screen at 24 months, which is a crucial step in the screening pathway for ASD. It is important to note that a positive M-CHAT is a screening indicator, not a clinical diagnosis of ASD; children with positive screens require comprehensive diagnostic evaluation. The clinical value lies in potentially flagging children earlier in the screening process for closer monitoring and, if later diagnosed, earlier intervention. However, it is crucial to emphasize the preliminary nature of these findings, which are derived from a retrospective design. Therefore, these results must be validated in prospective longitudinal cohorts that include gold-standard diagnostic confirmation before any definitive clinical recommendations can be made.

## Availability of Data and Materials

The datasets used and analyzed during the current study are available from the corresponding author on reasonable request.
